# Focus on the research utility of intravascular ultrasound - comparison with other invasive modalities

**DOI:** 10.1186/1476-7120-9-2

**Published:** 2011-01-30

**Authors:** Christos V Bourantas, Scot Garg, Katerina K Naka, Attila Thury, Angela Hoye, Lampros K Michalis

**Affiliations:** 1 Department of Cardiology, Castle Hill Hospital, Cottingham, East Yorkshire, UK; 2 Department of Cardiology, Medical School, University of Ioannina, Ioannina, Greece; 3 Department of Cardiology, Albert Szent-Gyorgyi Clinical Center, University of Szeged, Szeged, Hungary

## Abstract

Intravascular ultrasound (IVUS) is an invasive modality which provides cross-sectional images of a coronary artery. In these images both the lumen and outer vessel wall can be identified and accurate estimations of their dimensions and of the plaque burden can be obtained. In addition, further processing of the IVUS backscatter signal helps in the characterization of the type of the plaque and thus it has been used to study the natural history of the atherosclerotic evolution. On the other hand its indigenous limitations do not allow IVUS to assess accurately stent struts coverage, existence of thrombus or exact site of plaque rupture and to identify some of the features associated with increased plaque vulnerability. In order this information to be obtained, other modalities such as optical coherence tomography, angioscopy, near infrared spectroscopy and intravascular magnetic resonance imaging have either been utilized or are under evaluation. The aim of this review article is to present the current utilities of IVUS in research and to discuss its advantages and disadvantages over the other imaging techniques.

## Introduction

Utilization of conventional coronary angiography has certain limitations in the prognosis of coronary atherosclerosis, as the risk of experiencing a coronary event does not only depend upon the severity and extent of a lesion, but also on the size, histology and biological activity of the plaque. Some of these limitations can be addressed by intravascular ultrasound (IVUS) a modality which provides two dimensional (2-D) cross-sectional arterial images. In these images the lumen, outer vessel wall, plaque and stent can be identified and accurate measurements can be obtained. The fact that it provides reliable results in real time and it is widely available has rendered it a useful tool in clinical practice and research. Thus, though it is an expensive and time consuming procedure and caries a small risk of complications (mainly spasm but also the risk of embolism, thrombus formation and dissection), IVUS is often used to assess the severity of intermediate lesions, to guide treatment in high risk patients and complex lesions and to examine the final outcome after a percutaneous coronary intervention (PCI) [1,2].

Apart from its clinical applications, IVUS has also been proven a useful tool in research in the study of plaque evolution and in the evaluation of new interventional or pharmacological treatments. Recent developments in IVUS processing and especially the analysis of intravascular ultrasound radiofrequency (IVUS-RF) backscatter signal have provided further information regarding the composition and mechanical properties of the plaque and enhanced the role of IVUS in the study of atherosclerosis [3]. However, IVUS still has indigenous limitations such as the noise and the low axial resolution, which do not allow detailed visualisation of certain lumen and plaque characteristics. For these limitations to be addressed, alternative invasive modalities with different strengths and weaknesses have been developed such as angioscopy, optical coherence tomography (OCT), near infrared spectroscopy (NIRS) and intravascular magnetic resonance imaging (IV-MRI).

The aim of this review article is to discuss the advantages and disadvantages of IVUS over the other imaging techniques and highlight its value in research.

## I) Early application of IVUS imaging in research

The superiority of IVUS imaging over angiography were obvious from its initial steps [4,5]. To facilitate its application in research a number of tools were developed that provided fast and reliable IVUS processing and quantitative analysis [6-8]. This allowed the use of IVUS in numerous studies which helped us to explain the mechanisms of atherosclerotic process and affected the evolution in interventional cardiology. In the early stages of PCIs IVUS was used to elucidate the mechanisms of action of balloon angioplasty (arterial expansion and plaque fracture) and understand the causes of restenosis (vessel wall negative remodelling and intima proliferation) [9,10]. These data suggested the use of stents as it was believed that they would reduce the risk of restenosis. However, during the initial use of bare metal stents (BMS) high rate of acute and sub-acute stent thrombosis as well as restenosis were noted. IVUS imaging identified as predictors of sub-acute stent thrombosis the incomplete stent strut apposition, the asymmetrical stent expansion and the residual lumen narrowing and also showed that restenosis mainly occurs within the first 6 months post stent implantation [11]. To overcome these problems post stent dilation with larger balloons and higher pressures was recommended while research was driven towards the creation of advanced stent platforms and the development of drug eluting stents (DES) [12].

## II) Recent applications of IVUS imaging in research

### a) Study of plaque progression - vascular remodelling

Coronary angiography is unable to provide detailed information regarding plaque burden, as initially atherosclerosis may not cause luminal narrowing accommodating the evolving plaque in the vessel wall which expands outward (positive remodelling). On the other hand IVUS permits complete vessel visualisation and accurate assessment of the atherosclerotic burden and thus it appears more sensitive than quantitative coronary angiography (QCA) in detecting the progression of atherosclerosis [13]. The fact that high plaque burden is related to a higher risk of cardiovascular events has allowed IVUS measurements to be used as surrogate endpoints, instead of clinical endpoints, in trials that investigated the effect of several pharmacological treatments on plaque progression [14,15]. In this way IVUS imaging appeared a cost effective technique as it permitted studies to be conducted with a smaller number of patients and completed in shorter time interval.

Hence, today it is known that aggressive lipid-lowering therapy (with high doses of atorvastatin or rosuvastatin) induces plaque regression and that pioglitazone has a favourable effect on coronary atherosclerosis [16-20]. Similarly, the CAMELOT study used IVUS to show that amlodipine reduces plaque burden while the PERSPECTIVE study demonstrated that perindopril does not affect the progression of the atheroma [21,22]. In addition, serial IVUS examinations were implemented to study the effect of new drugs such as the reconstituted HDL (CSL-111), the dalapladip (a lipoprotein-associated phospholipase A_2 _inhibitor) and the pactimibe (a non-selective inhibitor of acyl-coenzyme A:cholesterol acyltransferase) on plaque development. In these studies it was found that all the new treatments had a neutral effect on plaque burden though dalapladip appeared to reduce the lipid core expansion [23-25].

The ability of IVUS to display both the plaque and the whole vessel wall provided us with an insight into the mechanisms, and the prognostic value of vascular remodelling. IVUS has been used to show that negative remodelling (defined as a shrinkage of the vessel wall at the lesion site) is more common in elderly patients and stable plaques, whilst on the other hand it appears that plaques with positive remodelling (defined as vessel wall expansion at the lesion site) contain more lipid-rich components, and are associated with an increased risk for acute coronary events [26,27].

**Figure 1:**
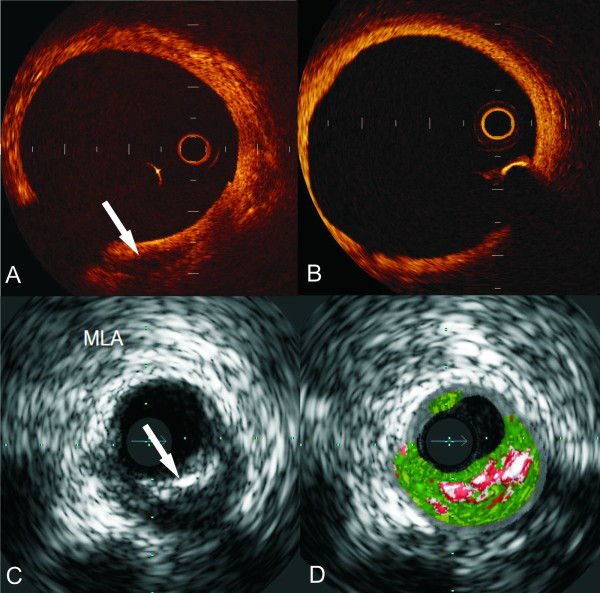
**OCT imaging may allow visualisation of the vessel wall behind a calcified plaque (A) but on the other hand often fails to fully visualise the arterial wall because of its poor penetration (B)**. The limitation of IVUS to identify the type and the extent of the plaque behind the calcium (arrow) (C) has been successfully addressed by IVUS-RF analysis (D).

Compared to other invasive imaging techniques IVUS is superior in quantifying changes in plaque volume and measuring vessel wall dimensions and thus is the preferable modality for assessing the effect of pharmacological treatment on coronary atherosclerosis. Angioscopy cannot be used to measure the plaque as it provides only imaging of the luminal surface and gives no data on the vessel wall. On the other hand, OCT although allows imaging of the atheroma, and more accurate computation of plaque volume in case of calcium deposits, since it lacks the shadowing artefacts, often cannot portray the whole vessel as it has poor tissue penetration (Figure 1). To address this drawback several new approaches have been proposed (e.g. spectral radar OCT, use of a parallel ultrasound beam, image processing techniques etc.) however, further development is necessary before these techniques can be used *in vivo *for the evaluation of vascular pathology [28].

### b) Characterisation of the type of the plaque - vulnerable plaque detection

It is well known that IVUS has limited capability in assessing plaque composition and detecting the features associated with plaque's vulnerability. To address these drawbacks analysis of IVUS radiofrequency backscatter signal has been proposed. This signal processing approach was validated using histopathologic findings as gold standard and it was found that it can identify the type of the plaque with high sensitivity, specificity and predictive accuracy [29,30]. This ability, the comprehensive images and the quantitative measurements that it provides allowed the broad use of IVUS-RF in the study of plaque development. Several IVUS-RF based studies have showed that the composition of the atheroma is affected by co-morbidities such as hypertension (increased fibrous plaques), diabetes mellitus (larger lipid cores and less fibrous plaques) or the presence of the metabolic syndrome (increased lipid rich plaques); while others demonstrated that therapy with statins has favourable effects, as it stabilizes the plaque by reducing its lipid component [31-33].

Kubo et al. used serial IVUS-RF imaging to investigate the natural evolution of non-obstructive plaques and showed that in contrast to fibrous and calficied plaques which remain unchanged the intimal thickening and thick cap fibroatheromas may evolve to thin cap fibroatheromas at 12 months follow-up [34]. Moreover, the PROSPECT trial used IVUS-RF to study the natural progress of atherosclerosis in 700 patients who presented with acute coronary syndrome. All patients underwent successful PCI and three vessel imaging with IVUS and IVUS-RF. At 3 years follow-up major adverse cardiac events occurred in 20.4% of the patients. After examining the imaging data at follow-up it was found that presence of a thin-cap fibroatheroma, plaque burden ≥70% and a minimum luminal area ≤4 mm^2 ^were associated with an increased risk for cardiovascular events.

However, although IVUS-RF appears reliable in characterising the type of the plaque and studying its evolution, it has limited capability in identifying several features which according to Naghavi et al. are associated with increased vulnerability (Table 1) [35]. Autopsy studies showed that a vulnerable plaque is mainly found in segments with positive remodelling, is infiltrated by macrophages and consists of a lipid rich core covered by a thin fibrous cap which is maybe disrupted. The fact that IVUS-RF has a reduced axial resolution (range: 100-200 μm) restricts its ability to identify some of these features (e.g. plaque disruption, infiltration of macrophages) and to measure the thickness of the fibrous cap [36]. To enhance the effectiveness of IVUS in detecting features associated with increased vulnerability several methodologies have been proposed such as palpography that measures the local strain of the plaque and contrast enhanced IVUS which in a single study was implemented to detect the vasa vasorm and increased neovascularization [37-39]. Though the first results appear promising, natural history and multicentre randomized interventional trials should be performed in order to establish their ability to identify high risk plaques.

**Table 1:** Histological features of vulnerable plaque - ability of invasive imaging modalities in identifying vulnerable plaque characteristics.

Criteria for defining a vulnerable plaque	Intravascular imaging modalities				
***Major Criteria***	***IVUS, IVUS-RF***	***OCT***	***Angioscopy***	NIRS*	IV-MRI*
Active inflammation	-	+	-	-	-
Thin fibrous cap	++	+++	++	-	+
Lipid core	+++	+++	-	++	++
Disrupted plaques	+	+++	++	-	-
Stenosis > 90%	+++	+	-	-	++
					
***Minor Criteria***					
Superficial calcified nodule	-	++	-	-	-
Yellow colour on angioscopy plaque	-	-	+++	-	-
Intraplaque haemorrhage	-	+	-	-	-
Endothelial dysfunction	-	-	-	-	-
Positive remodelling	+++	+	-	-	++

IVUS, intravascular ultrasound; RF, radiofrequency analysis; OCT, optical coherence tomography; NIRS, near infrared spectroscopy; IV-MRI, intravascular magnetic resonance imaging. Methods' ability to identify vulnerable plaque characteristics is graded as: unable (-), low capability (+), moderate capability (++) and high capability (+++). The evaluation of the methods that have not been used in clinical practice (indicated with *) was based on the in vitro and ex vivo studies.

OCT has emerged as a promising imaging modality for evaluating plaque composition and assessing plaque's vulnerability. The high resolution of OCT imaging allows the identification of lipid pools and in contrast to IVUS, detection of the internal and external elastic lamina [40,41]. Another advantage of OCT is the lack of shadowing artefacts, in case of calcium deposits that enables visualisation of the adjacent tissues. OCT also provides accurate quantification of fibrous cap thickness, allows reliable evaluation of cap disruption and erosion and is able to clearly visualise the presence and type of thrombus [36,42]. Finally, it has been speculated that OCT enables detection of macrophages infiltration, though this has not been evaluated in large scale trials [43]. A limitation of this modality is the restricted axial penetration that may impede the estimation of lipid pool dimensions, and identification of positive remodelling. To overcome this, combination of IVUS-RF and OCT imaging has been suggested [44].

Angioscopy, though not useful in characterising plaque's constitution, has been used for the identification of the high risk plaques as it can reliably demonstrate the presence and the type of thrombus and has similar sensitivity with IVUS in detecting plaque erosion and disruption [36]. In addition, it has been showed that the colour of the plaque depends on the thickness of the fibrous cap, with the yellow plaques having the histological characteristics of a vulnerable plaque (a thin fibrous cap covering a lipid-rich core) and white plaques being stable having thick fibrous caps [45]. However, routine use of angioscopy is severely limited as it requires full obliteration of the vessel and disruption of the coronary circulation and thus it cannot be used to visualize long coronary segments.

### c) Evaluation of drug eluting and bioabsorbable stents

IVUS is a useful tool in evaluating the efficacy of different invasive treatments, identifying predictors of in-stent restenosis and stent thrombosis and understanding the underlying mechanisms. Its ability to measure neointimal hyperplasia has allowed IVUS to be used in landmark studies which examined the effectiveness of DES over BMS [46,47]. It has also been implemented to assess the performance of new platforms (e.g. MAHOROBA study showed failure of the tacrolimus-eluting stent to prevent neointimal hyperplasia) and to compare the efficacy of treatment options for in-stent restenosis [48-50]. Moreover, IVUS provided an insight into some of the mechanisms associated with stent thrombosis in DES era and thus today it is known that stent underexpansion, residual reference segment stenosis, incomplete stent apposition and coronary dissection after DES implantation are associated with an increased risk for stent thrombosis and future major adverse cardiac events [51-53].

IVUS has also been implemented to test the safety profile, vessel wall reactions, and mechanical behavior of bioabsorbable stents. These stents, been constructed by a material which is gradually absorbed, are expected to not impair the image quality of the non-invasive imaging modalities and to have less late thrombosis rates. Several studies used IVUS to assess luminal dimensions and remodeling after bioabsorbable stent implantation [54-56]. The ABSORB found favorable results while the PROGRESS-AMS showed a significant late luminal loss after the implantation of a bioabsorbable magnesium stent fact that was attributed to recoil and neointimal hyperplasia [55,56]. In the ABSORB study a multitude of imaging modalities (QCA, IVUS, IVUS-RF, palpography, OCT, and computed tomographic (CT) coronary angiography) were implemented to study the behavior of the bioabsorbable everolimus-eluting stent. In this study IVUS and IVUS-RF appeared useful in measuring the luminal and plaque dimensions after stent implantation and assessing alterations in plaque constitution whereas palpography was used to evaluate changes in the deformability of the vessel wall caused by the stent absorption [57,58].

**Figure 2:**
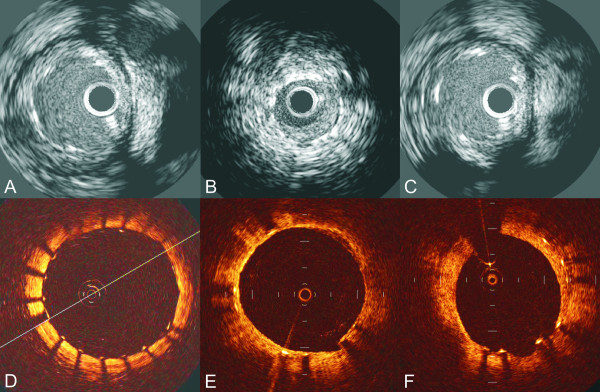
**IVUS images showing optimal stent expansion (A); neointima formation (B) and late stent malaposition 6 months after a DES implantation (C)**. OCT imaging allows not only assessment of stent expansion but also evaluation of stent struts coverage (absence of coverage (D) vs. complete coverage (E)) and measurement of neointimal hyperplasia (F).

OCT appears equally useful in assessing the efficacy of stents. An advantage of OCT is that it can accurately detect thrombus and vessel trauma (e.g. depth of dissections caused by balloon inflations and cuts in the atherosclerotic plaque made by the blades of cutting balloons) during invasive treatments and detect the presence (or absence) of stent struts coverage [59]. Furthermore, OCT is superior to IVUS in identifying incomplete stent apposition and stent malapposition but in contrast to IVUS it cannot assess the effect of vascular remodelling to late stent malapposition (Figure 2) [60,61].

Angioscopy has also been utilised, but in less extent, to assess the efficacy of invasive treatments. Comparing to IVUS, angioscopy allows more reliable evaluation of vascular trauma after PCIs and can be used to provide an insight into stent struts coverage after DES implantation [62]. On the other hand it fails to quantify stent struts coverage and luminal dimensions and cannot assess stent apposition and vascular remodelling.

### d) The role of heamodynamics on the atherosclerotic process

**Figure 3:**
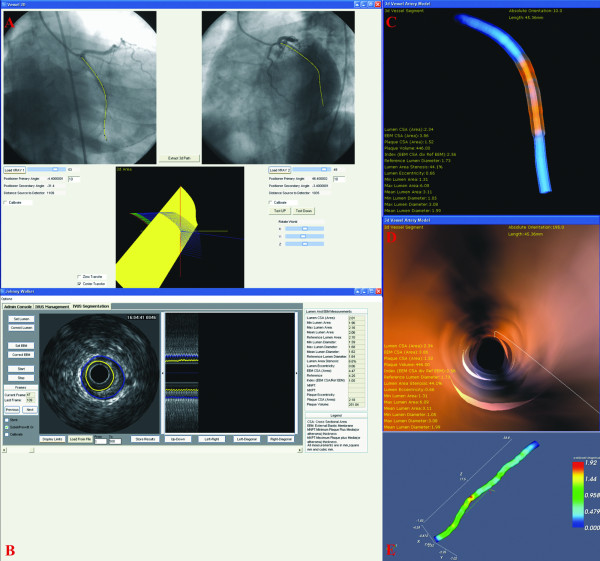
**3-D reconstruction of a coronary artery and blood flow simulation**. (A) Extraction of the IVUS path from biplane angiography; (B) semi-automated border detection of the IVUS images; (C) placement of the detected borders onto the catheter path and determination of their absolute orientation; (D) virtual endoscopy of the reconstructed vessel; (E) blood flow simulation into the final model and determination of the endothelial shear stresses.

Over the last decade IVUS has been combined with angiography for the reconstruction of coronary arteries [63,64]. The obtained models which currently constitute the state of the art in coronary 3-D imaging, provide fully and comprehensive arterial representation and have been used to investigate the role of local heamodynamics in the atherosclerotic process (Figure 3). Several investigators implemented these models to demonstrate that low and oscillatory shear stresses are associated with an increased risk for in-stent restenosis and atherosclerosis progression in native coronary segments and coronary bifurcations while recently, it was shown that low shear stresses not only act as an atherogenic factor but also promote the development of vulnerable plaque [65-68]. These studies have provided useful information and helped us to explain the regional localisation of atherosclerosis and understand the role of flow dynamics in plaque evolution and destabilisation [69].

### e) Future developments

**Figure 4:**
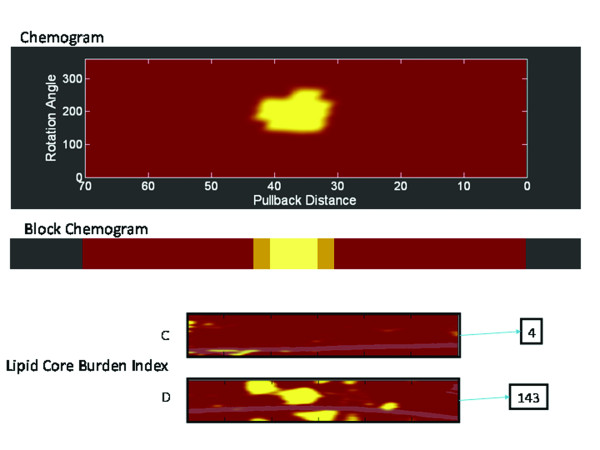
**The outputs from the Near-infrared spectroscopy device**. (A) The chemogram is a map of the measured probability of the presence of lipid core plaque from each scanned arterial segment and displays pullback position against circumferential position of the measurement in degrees. The device attributes a yellow colour to those regions with the highest probability of lipid core plaque, whilst red represents those with the lowest. In this example, a high probability of lipid core is detected at 30-40 cm proximal to the origin of the pullback. (B) A block chemogram provides a summary of the raw data from the chemogram and displays the probability that a lipid core is present for all measurements in a 2 mm block of coronary artery. The order of probability for the presence of lipid core plaque from highest to lowest is yellow, light brown, brown, and red. (C and D) The lipid core burden index, a numerical value, which ranges from 0-1000, gives an impression of the lipid content within the artery. The chemogram (C) has very little in the way of yellow regions, compared to the chemogram (D), reflecting this, the respective lipid core burden indexes are 4 and 143.

NIRS relies on the principle that different organic molecules absorb and scatter NIRS light to different degrees and at various wavelengths. The ability of NIRS to detect lipid core containing coronary plaques has been known for over a decade. However, only recently technology enabled the development of a catheter suitable for the human coronary artery. The device is attached to a console, which interprets the NIRS signals, and produces three outputs: the chemogram, the block chemogram and the lipid core burden index none of which require additional processing (Figure 4). A significant limitation of this modality is its inability to provide information regarding the dimensions and the morphology of the regions of interest. This limitation has been addressed by a new catheter that combines both a NIRS and a 40 MHz IVUS probe and enables co-localization of lipid core with structure. The feasibility of this hybrid modality has already been examined in-vivo and the first results appear promising for the future [70].

IV-MRI appears also to be a potentially useful technique for tissue characterisation [71]. Nevertheless, limitations, such as the increased size of the catheters and the risk of vessel wall heating, caused by the intense radiofrequency administration required for complete tissue characterisation and anatomic depiction, need to be addressed before this method can be used in clinical practice [72].

Finally, new non-invasive imaging modalities are likely to emerge in the near future as an attractive alternative in the study of atherosclerosis. The combination of positron emission tomography with CT imaging has already allowed the anatomic identification of nuclear tracers attracted by inflamed plaques, and thus this approach can be used for the detection of vulnerable plaques [73]. CT coronary angiography is another promising technique. Currently, it allows visualization of plaque and differentiation of calcified from non calcified atheroma. However, it cannot discriminate fibrous from lipid plaques and has limited capability in assessing plaque burden in de-novo and especially in stented segments [74,75]. Unfortunately, MRI imaging has low resolution which limits coronary visualization; hence further development is required before its implementation in the clinical and research arena.

## Conclusions

Through our review it is apparent that today there is a multitude of imaging modalities able to assess different morphological and functional features of the coronary arteries. Amongst these IVUS constitutes a valuable tool in research as it is the gold standard for studying plaque development and assessing the effectiveness of pharmacological treatments on plaque's evolution (Table 2). Though it is the most widespread invasive imaging modality it has significant intrinsic limitations being the considerable noise and the low axial resolution which does not allow detailed assessment of luminal morphology and meticulous visualisation of plaque characteristics. Thus, alternative intravascular imaging techniques have been introduced which provide additional information regarding the composition and vulnerability of the plaque and allow more accurate evaluation of stent deployment and struts' coverage. The combination of IVUS with these modalities, either as independent or as hybrid imaging, may allow in the future a more detailed insight into the mechanisms of plaque progression-regression and provide better evaluation of the different therapeutic strategies.

**Table 2:** Research utility of IVUS imaging. Advantages and disadvantages.

	IVUS imaging advantages	IVUS imaging disadvantages	Preferable modality
**Assessment of the effect of pharmacological treatment**	• Able to quantify changes in plaque volume • IVUS-RF allows identification and quantification of changes in plaque's composition	• IVUS-RF identifies with moderate sensitivity/specificity a change from lipid to a fibrous plaque	IVUS
**Remodelling assessment**	• Complete arterial wall visualisation	• Unable to identify accurately the outer vessel wall border in segments with calcified plaques.	IVUS
**Plaque characterisation**	• Complete vessel wall visualisation • IVUS-RF allows identification of the type of the plaque with good overall sensitivity and specificity	• IVUS-RF identifies with moderate sensitivity/specificity lipid and fibrous plaques	IVUS-RF and OCT
**Detection of vulnerable plaque**	• Accurate measurement of luminal dimensions, plaque area and remodelling • IVUS-RF allows identification of the type of the plaque with good overall sensitivity and specificity	• Limited axial resolution - unable to measure the fibrous cap • Moderate sensitivity in detecting thrombus and plaque disruption/erosion • Unable to detect macrophages or intraplaque haemorrhage	OCT and IVUS-RF or combination of different imaging modalities
**Assessment of invasive treatments**	• Reliable assessment of luminal, stent area and intima hyperplasia • Precise evaluation of stent expansion • Reliable evaluation of bioabsorbable stent recoil	• Limited capability in identifying vessel wall trauma (e.g. erosion, dissection) and thrombus • Incapable of assessing stent struts coverage	OCT or combination of OCT and IVUS
**Role of heamodynamics in atherosclerosis**	• Complete vessel visualisation - plaque characterisation • Multitude of automated methodologies that allows IVUS segmentation and fusion of IVUS and angiography	• Limited capability in detecting vulnerable plaque characteristics	IVUS

IVUS, intravascular ultrasound; RF, radiofrequency analysis; OCT, optical coherence tomography; NIRS, near infrared spectroscopy.

## Competing interests

The authors declare that they have no competing interests.

## Authors' contributions

CVB wrote the manuscript while the other authors involved in drafting and revised the manuscript for important intellectual content. All authors read and approve the final document.
